# Genito‐Pelvic Pain/Penetration Disorder Presenting as an Unconsummated Marriage Managed in Japanese Primary Care

**DOI:** 10.1002/jgf2.70177

**Published:** 2026-09-11

**Authors:** Hisashi Yoshimoto, Sayaka Ohsawa, Hiroki Isono, Satoko Komori, Shoji Yokoya

**Affiliations:** ^1^ Department of Family Medicine, General Practice and Community Health, Institute of Medicine University of Tsukuba Tsukuba Japan; ^2^ Kitaibaraki Center for Family Medicine Kitaibaraki Japan; ^3^ Research and Development Center for Lifestyle Innovation University of Tsukuba Tsukuba Japan; ^4^ Tsuchiura Rehabilitation Hospital Tsuchiura Japan; ^5^ Department of General Medicine HITO Medical Center Shikokuchuo Japan; ^6^ Takahashi Clinic Saitama Japan

**Keywords:** family medicine, genito‐pelvic pain/penetration disorder, primary care, sexual dysfunction, unconsummated marriage

## Abstract

**Background:**

Genito‐pelvic pain/penetration disorder (GPPPD) may present as unconsummated marriage and receive less attention when pregnancy is prioritized.

**Case:**

A 39‐year‐old woman and her husband presented after 5 years without penovaginal intercourse despite fertility treatment. Clinical history supported GPPPD. Primary care included biopsychosocial assessment, psychoeducation, and couple support. Stepwise care included paced breathing, pelvic floor relaxation stretches, lubricant, graded insertion, and vaginal dilation. Intercourse was achieved after 8 months, patient‐rated overall satisfaction rose from 0 to 85/100, and she had a child.

**Conclusion:**

Family physicians may support couples with GPPPD using biopsychosocial, stepwise care when specialist services are limited.

## 1 Introduction

Genito‐pelvic pain/penetration disorder (GPPPD) includes persistent difficulty with vaginal penetration, genito‐pelvic pain, penetration‐related fear or anxiety, and pelvic floor tightening [1]. Sexual pain may impair psychological well‐being and intimate relationships [2]. Unconsummated marriage is the inability of a heterosexual married couple to achieve penovaginal intercourse; vaginismus/GPPPD‐related conditions and erectile dysfunction are common causes [3]. Couples seeking pregnancy may first enter fertility care, where pregnancy becomes the dominant goal. Sexual health concerns are also difficult to raise in primary care [4, 5]. We report this case to highlight the role of primary care in recognizing and providing stepwise, couple‐oriented care for GPPPD presenting as unconsummated marriage when specialist services are not readily available.

## 2 Case Report

A 39‐year‐old woman presented with her husband because intercourse had never been possible despite repeated attempts during 5 years of marriage. She reported no sexual abuse, mental illness, or medication causing sexual dysfunction. Since their first attempts, penetration caused pain at the vaginal introitus, pelvic floor tightening, and marked anticipatory anxiety. She felt guilty toward her husband and pressured by her parents to become pregnant. The husband reported no erectile or ejaculatory difficulty. Despite a good relationship, they had discussed divorce if infertility treatment failed and intercourse remained impossible.

Six months before presentation, she had consulted a gynecologist for infertility. According to the couple, ovulation assessment, hormonal testing, hysterosalpingography, and semen analysis showed no major abnormality. Intrauterine insemination was offered to enable conception while intercourse remained impossible, but they received insufficient advice about intercourse difficulty. They sought our clinic because they regarded it as a family issue: “We thought a family medicine clinic might help.”

Based on persistent penetration‐related pain, anxiety, pelvic floor tightening, clinically significant distress for longer than 6 months, no reported organic gynecological cause, and no psychiatric or medication‐related cause, GPPPD presenting as unconsummated marriage was clinically diagnosed.

Because specialist psychosexual services were not readily available, management proceeded in primary care. The first visit included both partners; most follow‐ups were with the patient alone. Initial visits lasted about 30 min, then usually 5–10 min, within routine health‐insurance care. Visits were initially about every 3 months and later about monthly as common diseases were also managed. Initial care included biopsychosocial assessment, psychoeducation, reassurance not to rush intercourse, explanation of the pain‐anxiety‐tightening‐avoidance cycle, support for couple communication, and infertility‐related questions. Relaxation strategies included paced breathing and pelvic floor relaxation stretches, such as child's pose and a relaxed frog stretch.

**FIGURE 1:**
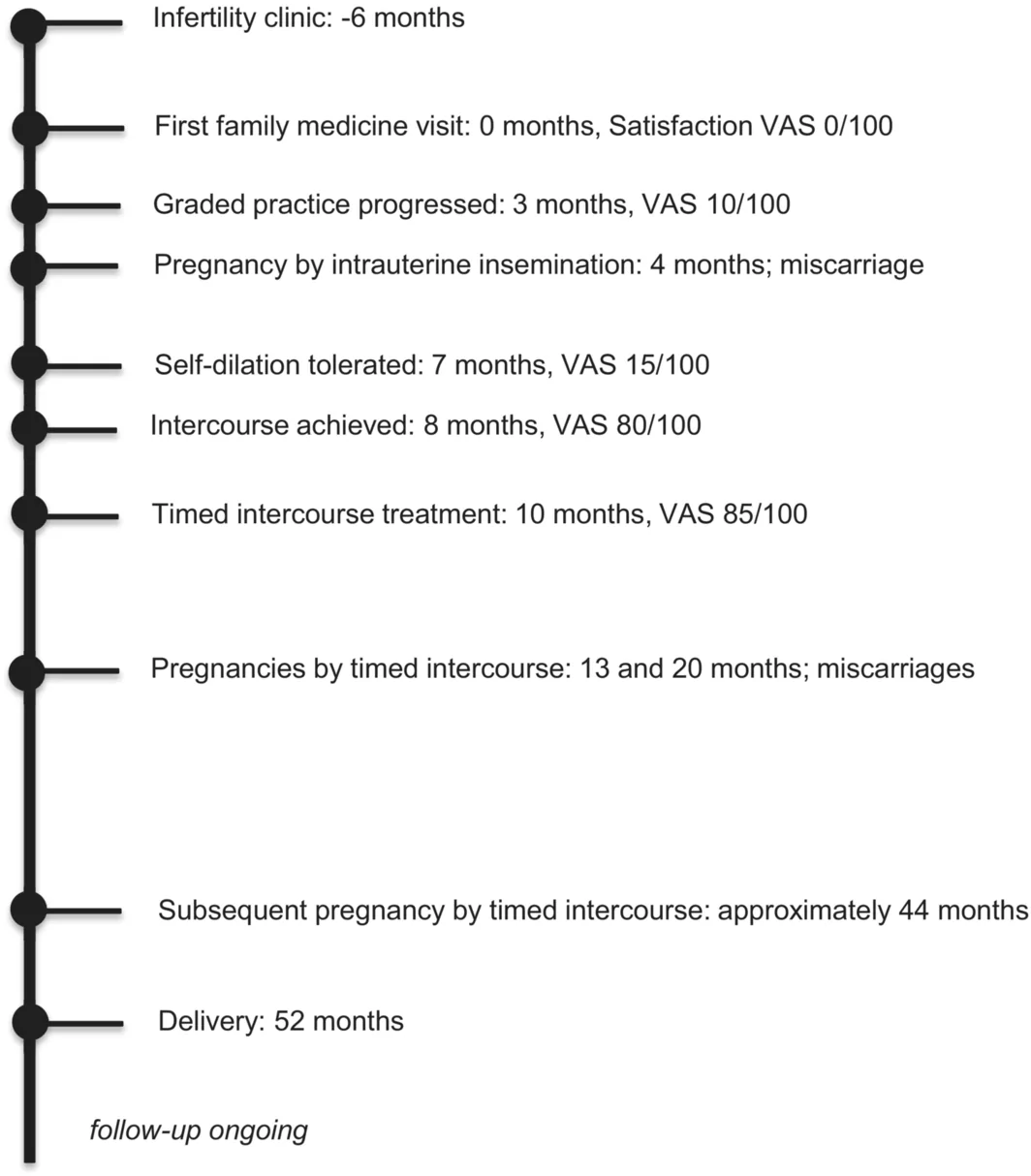
Clinical timeline and overall satisfaction visual analog scale (VAS).

The physician showed examples of commercially available vaginal dilators and advised gradual self‐insertion at a tolerable pace with lubricant, without forcing penetration. Advice also addressed sexual positions, foreplay, emotional intimacy, and vibratory stimulation. By the second visit, she reported insertion of two fingers and use of a dilator. Two months after presentation, gynecological examination became tolerable. While GPPPD care continued, intrauterine insemination resulted in pregnancy at 4 months, but the pregnancy ended in miscarriage of unknown cause. Because the couple wished to achieve intercourse as well as pregnancy, treatment continued; intercourse was achieved at 8 months with less fear. Infertility care then changed to timed intercourse. Overall satisfaction with her own and the couple's situation, rated on a 0–100 visual analog scale (VAS), increased from 0 to 85 (Figure 1). Timed‐intercourse pregnancies at 13 and 20 months ended in miscarriage. She conceived again by timed intercourse at approximately 44 months and gave birth at 52 months. At long‐term follow‐up, she reported reduced psychological distress and no recurrence of penetration difficulty. When consenting, she stated that familiar physicians should support couples with similar difficulties because such access makes consultation easier.

## 3 Discussion

This case highlights two clinical lessons. First, fertility treatment should not replace treatment of penetration‐related distress when couples wish to address both. Intrauterine insemination enabled conception but did not address distress about intimacy; intercourse itself remained a meaningful outcome.

Second, family physicians can provide permission to discuss sexual concerns, clarify symptoms, encourage evaluation for organic causes, provide psychoeducation, support communication, and coordinate referral. The intervention used the Permission, Limited Information, and Specific Suggestions components of the PLISSIT (Permission, Limited Information, Specific Suggestions, Intensive Therapy) model [6]. Table 1 summarizes their integration into routine primary care. Published treatment courses vary: a structured program achieved intercourse in 96% after a mean of four sessions [7], therapist‐aided exposure in 89%, with 90% of successful cases within 2 weeks [8], and brief psychosomatic care in 64% by 6 months and 72% by 14 months [9]. The 8‐month course here occurred through intermittent routine primary care; overlapping treatment components do not imply equivalent efficacy.

**TABLE 1:** PLISSIT components and family medicine functions in this case.

Component	General meaning	Clinical response in this case	Family medicine/implementation function
Permission	Create a safe space to discuss sexual concerns	At the first visit, both partners discussed intercourse difficulty as a family and marital concern; later visits were mainly with the patient alone while the couple's shared goals remained explicit	The physician legitimized a sensitive concern as appropriate for consultation and maintained a nonjudgmental, couple‐oriented perspective
Limited information	Provide focused information about the condition and contributing factors	The physician explained GPPPD and the cycle of pain, anxiety, pelvic floor tightening, and avoidance	Shared information helped both partners reframe the difficulty as a recognized clinical problem, reducing isolation, self‐blame, and mutual blame
Specific suggestions	Offer practical strategies tailored to the patient	Stepwise advice included intimacy, foreplay, paced breathing, pelvic floor relaxation stretches, lubricant, graded insertion, and vaginal dilators	The physician translated specialist information into manageable steps, promoting understanding, minimizing confusion, and presenting suggestions as a shared care plan rather than pressure from one partner
Intensive therapy/referral	Referral to specialist treatment when needed	No specialist psychosexual therapy or pelvic floor physical therapy was readily available; instead, stepwise support continued in primary care	No separate specialty program was used; care was integrated into routine health‐insurance primary care visits. The first two visits were about 30 min, later visits were usually 5–10 min and longer when miscarriage was discussed, and intervals shifted from about every 3 months to about monthly as common primary care problems were also managed

This case has limitations. The family physician did not perform a pelvic examination or review gynecological records. Although an overall satisfaction VAS and clinical milestones were recorded, validated symptom‐specific measures were not used. Baseline sexual knowledge was not formally assessed, so its role in the difficulty or response to psychoeducation is unknown. Care was individualized rather than delivered through a standardized sex‐therapy program, limiting causal interpretation and reproducibility. Nevertheless, primary care may provide an accessible entry point when sexual, reproductive, and family concerns overlap.

## 4 Conclusion

Family physicians should consider GPPPD in couples with unconsummated marriage and ask whether they wish to address fertility, penetration‐related distress, or both.

## Author Contributions

**Hisashi Yoshimoto:** conceptualization, resources, investigation, writing – original draft. **Sayaka Ohsawa:** writing – review and editing, conceptualization. **Hiroki Isono:** writing – review and editing, conceptualization. **Satoko Komori:** writing – review and editing, conceptualization. **Shoji Yokoya:** writing – review and editing, conceptualization.

## Funding

The authors have nothing to report.

## Ethics Statement

Ethics review for this anonymized case report was waived by the institution.

## Consent

Written informed consent for publication was obtained from both the patient and her husband.

## Conflicts of Interest

The authors declare no conflicts of interest.

## Data Availability

The authors have nothing to report.
